# Effects of Sea-Ice Persistence on the Diet of Adélie Penguin (*Pygoscelis adeliae*) Chicks and the Trophic Differences between Chicks and Adults in the Ross Sea, Antarctica

**DOI:** 10.3390/biology12050708

**Published:** 2023-05-12

**Authors:** Deborah Maccapan, Giulio Careddu, Edoardo Calizza, Simona Sporta Caputi, Loreto Rossi, Maria Letizia Costantini

**Affiliations:** 1 Department of Environmental Biology, Sapienza University of Rome, Via dei Sardi 70, 00185 Rome, Italy; 2 CoNISMa, National Inter-University Consortium for Marine Sciences, Piazzale Flaminio 9, 00196 Rome, Italy

**Keywords:** *Pygoscelis adeliae*, trophic ecology, isotopic niche, sea-ice break-up, feeding strategies, stable isotopes, krill

## Abstract

**Simple Summary:**

The Adélie penguin, *Pygoscelis adeliae*, is one of the most abundant predators in the Antarctic. Its survival depends on numerous factors, including the presence of prey, closely related to sea ice. By changing sea-ice dynamics, climate change could affect its diet and recruitment. The aim of this study is to investigate, by means of stable isotope analysis of their faeces, penguin chicks’ diets in four colonies characterised by differing sea-ice persistence. In order to investigate any differences in diet between chicks and adults, the faeces of the latter were also collected and analysed. The study found that the contribution of krill to the chicks’ diets is generally greater than that of fish but that the proportion depends on the spatiotemporal variability of sea-ice dynamics. In addition, where sea ice was more persistent, the contribution of fish was lower in chicks than in adults. The less negative δ^13^C values of adults than chicks suggest that adults catch prey inshore for themselves and offshore for chicks. The expected variations in sea-ice dynamics and thus prey availability due to climate change might therefore modify the penguins’ diet and thus the role of this dominant endemic species in the Antarctic food web.

**Abstract:**

In Antarctica, prey availability for the mesopredator Adélie penguin, *Pygoscelis adeliae*, depends on sea-ice dynamics. By affecting cycles of sea-ice formation and melt, climate change could thus affect penguin diet and recruitment. In the light of climate change, this raises concerns about the fate of this dominant endemic species, which plays a key role in the Antarctic food web. However, few quantitative studies measuring the effects of sea-ice persistence on the diet of penguin chicks have yet been conducted. The purpose of this study was to fill this gap by comparing penguin diets across four penguin colonies in the Ross Sea and evaluating latitudinal and interannual variation linked to different sea-ice persistence. Diet was evaluated by analysing the δ^13^C and δ^15^N values of penguin guano, and sea-ice persistence by means of satellite images. Isotopic values indicate that penguins consumed more krill in colonies with longer sea-ice persistence. In these colonies, the δ^13^C values of chicks were lower and closer to the pelagic chain than those of adults, suggesting that the latter apparently catch prey inshore for self-feeding and offshore for their chicks. The results indicate that sea-ice persistence is among the principal factors that influence the spatiotemporal variability of the penguins’ diet.

## 1. Introduction

Rising global temperatures represent a major threat to ecosystems since they induce alterations in species composition and interactions within communities [1,2]. Antarctic ecosystems are extremely vulnerable to temperature variations because they interfere with seasonal sea-ice dynamics [3,4,5]. The cycles of formation and loss of sea ice, the dominant abiotic element to which most Antarctic organisms are adapted, strongly influence the aquatic primary productivity that fuels the continent’s biodiversity [5,6]. During spring, before the seasonal sea-ice break-up, primary productivity is mostly confined to sympagic unicellular algae growing on the underside of the sea ice [7,8]. During summer, the sea-ice break-up and melt release large amounts of these algae into the water column, along with micronutrients that, under conditions of adequate light radiation, favour the growth of phytoplankton [9,10]. The ecology and reproductive success of Antarctic animals are closely linked to this marked seasonal variation in sea ice, which causes spatiotemporal variation in resource availability [11] and thus food web structure [8,12,13]. The Adélie penguin (*Pygoscelis adeliae*; Hombron and Jacquinot, 1841) is an endemic Antarctic species and one of two penguin species living in the Ross Sea [14,15]. It is a pack-ice species that feeds under pack ice and coastal fast ice, as well as in open ice-free waters [16,17,18]. In the Ross Sea region, Adélie penguins feed mainly on krill belonging to the *Euphausia* genus and on the Antarctic silverfish *(Pleuragramma antarctica*) and bald rockcod (*Pagothenia borchgrevinki*)*,* whose availability has been found to be associated with sea-ice dynamics [19,20,21,22,23,24,25,26,27]. To a lesser extent, they feed on amphipods, other fish species [24,28,29] and, more rarely, on cephalopods in late summer [23,27,30].

Adults reproduce during the summer. After hatching, they feed their chicks, which are unable to catch prey on their own, by regurgitation for about three months [31,32]. During the breeding season, parents have a restricted foraging range since they need to return to the nest to feed their chicks [22,33,34]. The chick-rearing phase includes guard and crèche periods [32,35,36]. During the guard period, while one parent feeds and guards the chicks, the other reaches the sea to find food for itself and its offspring. During the crèche phase, chicks are left unattended and begin to form groups of chicks independent of the nest, while both parents search for food to cope with the increased nutritional needs of their offspring [37,38,39]. Their survival and growth are closely linked to their parents’ ability to provide them with the most profitable resources under a range of environmental conditions. In the Ross Sea region, Adélie penguins feed primarily on krill and pelagic and cryopelagic fish [23,24,27], whose availability closely depends on sea-ice dynamics [19,22,31,40]. However, studies indicate a variety of feeding patterns: some report greater consumption of krill in periods with no or low sea-ice coverage [23,41], and others in periods or areas with greater sea-ice coverage [42,43]. In addition, although the relationship between sea-ice dynamics and both demographic parameters and adult diets has been highlighted [18,41,44,45], there are few studies that provide quantitative measures of the effect of sea-ice persistence on the proportional consumption of prey items by chicks or on the trophic differences between chicks and adults. This requires attention, as future changes in the extent and persistence of sea-ice coverage could affect the recruitment of this species. Indeed, both early and delayed sea-ice break-up increase adult foraging energy costs, in the first case due to the low availability of resources, in the second due to the longer journey from the colony to the foraging areas at sea [23,41,46,47].

The purpose of our research was to fill these gaps, and to this end we used Stable Isotope Analysis (SIA) of carbon (^13^C/^12^C) and nitrogen (^15^N/^14^N) to analyse the diet of Adélie penguin chicks in four colonies in two breeding seasons differing in terms of the timing of seasonal sea-ice break-up in the Ross Sea. We also compared the diet of chicks with that of adults along the latitudinal break-up gradient in the year with the earlier sea-ice break-up, associated with anomalous warmer temperatures.

In recent years, SIA has been increasingly applied to the diet, trophic relationships and foraging areas of seabirds, including the Adélie penguin [25,48,49,50,51,52]. Together with body tissues, which may be difficult to collect and can be limited in terms of the number of carcasses available in situ, the isotopic signatures of faeces have also been used [25,53]. Faeces are a low-cost and readily available biological matrix. This allows us to reconstruct the short-term diet (3–4 days prior to sampling) of a mass of individuals with a non-invasive method. In this case, the differences between consumers and food resources are negligible [25,54], since faeces consist of food that is ingested but not assimilated [55,56].

Since δ^13^C values vary considerably between the different types of primary producer [8,13,57], the δ^13^C values of faeces make it possible to trace the main trophic chain from which the consumers derive their energy [25,56]. As pelagic phytoplankton have lower δ^13^C values than sympagic algae [8,12,13], they can also be used for coarse-scale determination of the penguins’ foraging sites, differentiating between offshore and inshore feeding [43,50]. The δ^15^N values increase predictably with trophic level [58] and can thus be used to estimate the consumer’s trophic position [36,58].

By combining the SIA of faeces with satellite image analysis, in this study we quantified the influence of sea-ice persistence on the penguins’ trophic niche and diet. Since population decline due to variation in the concentration and extent of sea ice has been both predicted and observed by studies of recruitment rate and chick body condition [46,59,60,61,62], our aim was to provide quantitative estimates of the effect of changing sea-ice persistence, measured in days, on the proportional contribution of various food sources to the penguin chicks’ diets. We expected chicks in colonies and years characterised by longer sea-ice persistence to have lower δ^15^N values, indicating lower consumption of fish [43,50,63]. Since sea-ice persistence obliges adults to undertake long field trips to reach favourable foraging areas at sea [23,34], and penguin species tend to partially digest their food during transport [64,65], we also expected differences in diet between chicks and adults. Evaluating chicks’ diets under variable sea-ice conditions will help understand the causes of the Adélie penguin’s decline and could also help predict possible cascading effects on the Antarctic food web, about one third of whose entire population is concentrated in the Ross Sea, the largest marine protected area in the world [66,67].

## 2. Materials and Methods

### 2.1. Study Area

**Figure 1:**
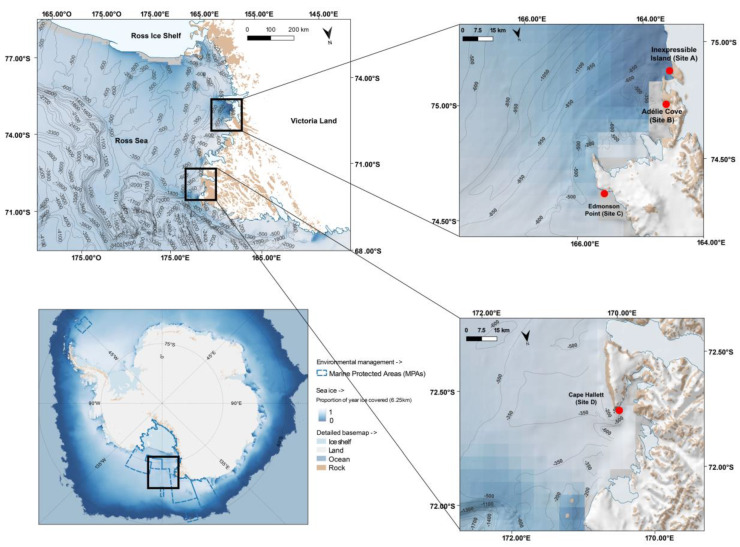
Map of Adélie penguin breeding colonies in the Ross Sea, where faeces samples and their main prey (krill and fish) were collected. The map was produced with Quantarctica V3 (http://quantarctica.npolar.no/about.html (accessed on 13 February 2023)) using the Landsat Image Mosaic of Antarctica (LIMA) satellite layer (US Geological Survey [68]). The map in the lower left shows the proportion of the year when the surface is covered in ice [69].

The study was carried out in mid-summer at four sites in the coastal area of the Ross Sea, Antarctica (Figure 1), classified as Important Bird Areas (IBA, [15]). From South to North, they were: Inexpressible Island (Site A), Adélie Cove (Site B), Edmonson Point (Site C) and Cape Hallett (Site D).

Inexpressible Island (74°54′01″ S, 163°43′02″ E) is located in Terra Nova Bay and it is bounded to the East by Hells Gate and Evans Cove and to the West by the Nansen pack ice. On the eastern side of the island there is a polynya, an area of open water surrounded by sea ice for several months of the year [70,71]. This area is home to various species of seabirds, including around 24,450 breeding pairs of Adélie penguins [15]. Adélie Cove (74°45′51″ S, 164°00′35″ E), located in Victoria Land on the Northern Foothills coast of Terra Nova Bay, hosts a colony of Adélie penguins with approximately 11,234 breeding pairs [15]. Edmonson Point (74°19′32″ S, 165°05′44″ E) is located at the foot of Mount Melbourne and overlooks Wood Bay. This site hosts an Adélie penguin colony consisting of about 1890 breeding pairs [15]. Cape Hallett (72°19′13″ S, 170°13′31″ E), a promontory located on the northern end of the Hallett Peninsula on the Borchgrevink coast in northern Victoria Land, a long distance from the polynya, hosts on average 42,628 breeding pairs of Adélie penguins [15].

The sampling activities were carried out in the crèche phase (between 17 January and 3 February 2017 and between 20 and 28 January 2018) during the 32nd and 33rd expeditions of the PNRA (Italian National Antarctic Research Program). The mean monthly air temperatures recorded in the study area in the 2016/2017 Antarctic summer (i.e., from December 2016 to February 2017) were −3.58 ± 1.16 °C, quite high compared to both those recorded during the same months of the Antarctic 2017/2018 summer (−5.71 ± 1.09 °C) and those recorded from 1987 to 2018 (SCAR MET-READER Database, www.scar.org (accessed on 23 January 2023), surface temperatures from the Mario Zucchelli, McMurdo and Scott Base stations). This trend has also been recorded on a larger scale in the broader Antarctic region (Copernicus Climate Change Service (C3S) Climate Bulletin, 2018, https://climate.copernicus.eu/ESOTC/2018 (accessed on 23 January 2023)).

### 2.2. Sampling Procedures

Faeces were collected in 15 mL sterile Falcon tubes using a spatula. In each colony, the chick faeces were collected from the crèche, while adult faeces samples were collected from the shoreline where adult penguins emerged from the water to reach the colony. In order to identify chicks and avoid pseudo-replication, the sampling entailed observing resting individuals and rapidly collecting freshly released faeces. Samples of fresh faeces were collected at a minimum distance of approximately 5 m from each other (one for each crèche). This allowed us to classify each sample as an independent observation (each sample was assigned to a different individual). In 2018, due to the low accessibility to the shore due to the presence of sea-ice blocks, it was not possible to obtain an adequate number of adult faeces samples to make comparisons.

Where possible, in order to assess site-specific diet, penguin preys were collected from the vomited undigested food of adult penguins found near the crèches. Undigested krill was recovered from vomit at all penguin colonies in both years, except at Cape Hallett in 2018, and was analysed separately to account for the isotopic values of the two site-specific species. Regarding fish, samples of *Pleuragramma antarctica* were found at Site A and Site C and were pooled, since their isotopic values did not vary. Since no specimens of the cryopelagic fish *Pagothenia borchgrevinki* were sampled in this study, its isotopic values were obtained from the literature. Samples of other potential preys, which included amphipods, the benthic fish *Trematomus bernacchii* and the benthic-pelagic *Chionodraco hamatus* [23,27], were collected during our sampling activities in the Ross Sea. Since in the study area cephalopods are a rather rare prey item in the Adélie penguins’ diet, and strictly in late summer [23,27,30], they were not considered in this population diet assessment.

Samples were stored in refrigerated containers until transport to the Mario Zucchelli Italian Research Station, enumerated and stored at −20 °C until transport to Italy for isotopic analysis.

### 2.3. Laboratory Procedures and Stable Isotope Analysis

In order to reduce any alteration during the nitrogen isotopic analysis, samples of faeces were cleaned by removing the residues of uric acid, rich in inorganic nitrogen, with a scalpel [54]. Samples of krill and amphipods were not acidified to remove carbonates before carbon isotopic analysis since each specimen was stripped of the exoskeleton and only the abdominal muscle was analysed. For *P. antarctica, T. bernacchii* and *C. hamatus*, one sample of dorsal muscle was taken from each fish [72].

The samples of faeces, krill, amphipods and fish were separately freeze-dried (−45 °C) for 24 h and pulverised to a fine homogeneous powder using a ball mill (Mini-Mill Fritsch Pulverisette 23: Fritsch Instruments, Idar-Oberstein, Germany). Aliquots of 1.0–1.5 mg were then weighed and pressed into tin capsules for the Stable Isotope Analysis. Samples were analysed using an Elementar Vario Micro-Cube elemental analyser (Elementar Analysen Systeme GmbH, Langenselbold, Germany) coupled with an IsoPrime100 continuous flow mass spectrometer (Isoprime Ltd., Cheadle Hulme, UK). Each sample was analysed twice, and values were averaged. Carbon (C) and Nitrogen (N) isotopic signatures were expressed in δ units (δ^13^C; δ^15^N) as parts per-thousand (‰) deviations from international standards: Vienna Pee Dee Belemnite (PDB) for C and atmospheric N_2_ for N. Isotopic ratios were computed in accordance with the Equation (1)

δX (‰) = [(*R*sample/*R*standard) − 1] × 1000 (1)

where X is the Carbon or Nitrogen isotope and *R* is the heavy-to-light isotope ratio of the respective element (^13^C/^12^C; ^15^N/^14^N). Results were calibrated to International Atomic Energy Agency reference materials (IAEA-CH-3, IAEA-CH3 and USGS24 for δ^13^C; IAEA-N1, IAEA-N2 and USGS25 for δ^15^N). Caffeine IAEA-600 (C_8_H_10_N_4_O_2_) was used as the internal laboratory standard (δ^15^N = 1.00‰ and δ^13^C = −27.77‰). Measurement errors associated with the linearity and stability of the mass spectrometer were typically smaller than 0.05‰, while the standard deviation of repeated measurements of lab standard material (one replicate every 10 analyses) was ±0.02‰ for δ^13^C and ±0.07‰ for δ^15^N.

### 2.4. Data Analysis

#### 2.4.1. Sea-Ice Persistence through Satellite Image Analysis

To assess sea-ice persistence, high-definition Landsat-8 OLI (Operational Land Imager) and TIRS (Thermal Infrared Sensor) satellite images, freely available on the USGS website (http://www.usgs.gov/ (accessed on 23 January 2023)), were analysed. For each year and colony, the date of sea-ice break-up was calculated (Videos S1 and S2). Each colony was considered free of sea ice when the fast ice extended less than 1 km from the shoreline to its seaward edge. The sea-ice edge was defined as the boundary between the fast ice and open water. In the absence of complete sea-ice break-up, the date with the minimum distance between the colony and the edge of the fast ice was considered. The difference in sea-ice persistence between colonies and between sampling years was calculated as the number of calendar days elapsing from the date of the earliest sea-ice break-up, which was observed at the polynya (Site A) on 27 September 2016. The delay in sea-ice break-up observed in 2017/2018 at each colony with respect to 2016/2017 was also calculated (Table 1).

Since the presence of the polynya is known to affect the break-up and reformation of sea ice on Inexpressible Island, with anomalies over time, the day of sea-ice break-up in this colony was also determined in accordance with Mengxi et al. [73].

#### 2.4.2. Isotopic Niche Analysis and Mixing Models

Population-wide niche metrics were applied to adult and chick isotopic data using stable isotope Bayesian ellipses in R (SIBER) [74,75]. Nitrogen range (NR) and carbon range (CR) were both calculated as the Euclidean distance between the highest and lowest respective isotopic values (δ^15^N, δ^13^C). NR indicates the population’s degree of omnivory, and CR gives information about the range of basal resources consumed [74]. The bi-dimensional isotopic niche space of each population was calculated as the total area (TA) and sample-size corrected standard ellipse area (SEAc). The TA encompasses all specimens and provides the total niche space occupied by each population, while the SEAc encompasses the core (around 40%) of the isotopic observations within each population and is less sensitive to sample size and isotopic outliers [75]. Bayesian Standard Ellipse Areas (SEAb) were calculated based on 1000 posterior draws with ‘uninformative’ priors and were used to evaluate the overlap of the chick and adult isotopic niches at each colony (SIBER analysis) [75]. Niche overlap was expressed as the proportion of the chicks’ isotopic niche that was encompassed within the isotopic niche of adults and was analysed in relation to sea-ice persistence. The values are reported in a density plot with 95, 75 and 50% credibility intervals. Before running mixing models for the quantification of the penguin diet, the possible contribution of each resource and the adequacy of the mixing spaces of consumers and resources were evaluated and validated using simulated mixing polygons (Figure S1). In the latter model, the greater the isotopic distance of the resource from the consumer, the lower the probability that it will actually be consumed [76,77]. Based on these results, all consumer points outside the 95% mixing region were excluded from the mixing models [76]. In order to quantify the diet of Adélie penguin adults and chicks, a Bayesian mixing model with ‘uninformative’ priors, returning outputs as probability distributions, was applied using the MixSIAR package [78,79]. The output of the model is a density function distribution of plausible proportions, whose central tendencies (mode, mean, median) and credibility intervals’ upper and lower limits (CI: 50%, 75%, 95%) reveal the range of contributions of each resource to the penguins’ diet [80]. Specifically, in this study, we report the mean and 95% credibility intervals.

The model required three inputs: (1) the isotopic signature of the target consumer; (2) the isotopic signatures (mean and standard deviation) of each of its potential food sources; and (3) the Trophic Enrichment Factor (TEF, also referred to as the discrimination factor), i.e., the difference between a consumer and its diet. Krill was characterised by greater spatial isotopic variability than the other prey here considered [51]. Thus, to take account of any spatial difference between krill (*E. superba* and *E. crystallorophias*), and to improve the accuracy of the Bayesian mixing models for the comparison between penguin colonies, we used site-specific krill isotopic values.

Since the isotopic signatures of the fish species are stable in space and time in the Ross Sea Region [21,50,52,81,82,83,84], the mean δ^13^C and δ^15^N values of each sampled species were used in the input model. The δ^13^C and δ^15^N values of amphipods were averaged, as they did not differ significantly between colonies and were consistent with those reported in the literature for the Ross Sea [13,29]. The TEF of faeces was considered equal to 0 for both C and N isotopes [54]. Indeed, the faeces consist largely of organic material not metabolised by the organism, and therefore fractionation is limited or absent [54,85].

#### 2.4.3. Data and Statistical Analysis

The δ^13^C and δ^15^N values of the faeces and prey (krill and fish) of the penguins were tested, respectively, for normality and homogeneity with the Shapiro-Wilks test and the Levene test. Given the normality of the data, parametric tests were used for all comparisons. Differences between prey species and between chicks and adults in terms of δ^13^C and δ^15^N values were tested using one-way ANOVA and Tukey post hoc comparisons. Two-way ANOVAs were also performed to test the effects of site, year and their interaction on the δ^13^C and δ^15^N values of (1) krill and (2) chick faeces. The results were considered significant when the *p* value was less than 0.05.

To ensure that the isotopic differences between the penguins could be ascribed to the different proportions of krill and fish in their diet and not to krill’s acknowledged space-time isotopic variability [86,87], before comparisons, the isotopic signatures of chick and adult faeces were standardised by subtracting the site- and year-specific isotopic values of krill.

We used the proportional consumption of each resource, obtained from mixing models, to calculate the trophic niche width of Adélie penguin chicks using the Shannon-Wiener diversity index (Hs). The linear regression model was used to test the relationship between sea-ice persistence and the variety of the penguin chick diet (Hs).

Beta regressions were used to test the relationship between sea-ice persistence and the percentage of krill, pelagic fish and amphipods in the Adélie penguin chicks’ diet. All statistical analyses were performed using R version 3.6.2 [78]. The beta regression models were run with the “betareg″ R-package [88].

## 3. Results

### 3.1. Sea-Ice Persistence

The timing of seasonal sea-ice break-up followed a latitudinal gradient, from the southern site (Site A), near the polynya, to the northern site (Site D), which was characterised by the longest sea-ice persistence. The earliest sea-ice break-up occurred at Site A in 2016/2017 (the date being used as a reference; Table 1). It occurred later at all sites in 2017/2018 with respect to 2016/2017, but the delay was greater in the southern sites than in the northernmost one. The dates of sea-ice break-up, together with the differences in sea-ice persistence between colonies and between years (measured in days), are reported in Table 1. Extreme weather conditions in Site A in 2017, especially katabatic winds and currents, led to recurring sea-ice break-up and formation. Two main break-up events occurred, one in September and one in October, followed by reformation, which caused anomalous extensive ice formation and persistence in December 2017. According to Mengxi et al. [73], during the first event, characterised by low temperatures and wind speeds, sea ice broke up and accumulated near the northernmost part of the bay. In the second event, the strong variations in wind speed and direction (from northwest to south) and the relatively low temperature contributed to the transport of ice to the coast, where it accumulated and was compressed, persisting until December [73].

### 3.2. Comparisons of Penguin Prey

Significant differences in δ^13^C and δ^15^N values were found between prey species, except between benthic and benthic-pelagic fish (*T. bernacchii* vs. *C. hamatus*), while differences between amphipods and krill were seen only in δ^15^N values (Table 2; one-way ANOVA, F_4,57_ = 191.66 and 225.60, *p* < 0.001, for δ^13^C and δ^15^N, respectively, and Tukey post hoc comparisons).

More specifically, the δ^13^C and δ^15^N values of the silverfish *P. antarctica* were higher than those of krill, except for δ^13^C in site C. The δ^13^C values of *T. bernacchii* and amphipods were higher than those of the other prey. The δ^15^N values of fish were higher than those of crustaceans, and those of *T. bernacchii* and *C. hamatus* were the highest of all fish (Tukey post hoc comparisons, *p* < 0.05).

The δ^13^C and δ^15^N values of krill differed between sites (Table 2 and Table 3). Notably, in site C, the δ^13^C and δ^15^N values of krill were much higher than in the other sites (Table 3; two-way ANOVA, F_3,14_ = 4.05 and F_3,14_ = 10.47, respectively; Tukey post hoc comparisons, *p* < 0.05 in all cases). In addition, the δ^15^N values of krill in both site A and site B were lower in 2018 than in 2017 (Table 3; two-way ANOVA, site-year interaction, *p* < 0.001, associated Tukey post hoc comparisons, *p* < 0.05 in both cases).

### 3.3. Comparisons of Chick Faeces between Years and between Colonies

**Figure 2:**
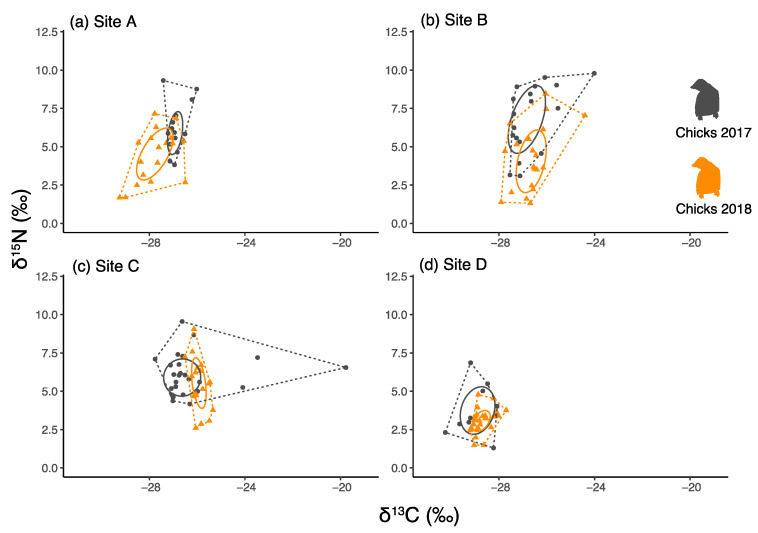
Isotopic niche biplots of Adélie penguin *(Pygoscelis adeliae)* chicks sampled in two consecutive years (2017 and 2018). Each circle/triangle represents a sample. The polygons represent the TA and the ellipses the SEAc. The 2017 chicks are represented in black and the 2018 chicks in orange. The letters indicate the colonies: (**a**) Site A: Inexpressible Island, (**b**) Site B: Adélie Cove, (**c**) Site C: Edmonson Point, (**d**) Site D: Cape Hallett.

Both the δ^13^C and δ^15^N of chick faeces differed among colonies (Table 1 and Table 3, two-way ANOVAs). Non-significant interaction between Site and Year was observed in the δ^13^C and δ^15^N values of chicks (Table 3). On average, δ^15^N values were lower in 2018 than in 2017 (Figure 2, Table 1 and Table 3), but in pairwise comparisons the difference was statistically significant only in the two southern sites (A and B; Tukey post hoc comparisons, Table S1).

δ^13^C values did not differ significantly between years in any colony (Table 3; Tukey post hoc comparisons always n.s.). The isotopic niche was considerably narrower in 2018 in the northern sites (C and D), but not in the southern ones (SEAc; Table 1).

### 3.4. Comparisons between Chicks and Adults

In 2016/17, the δ^13^C values were significantly lower in chicks than in adults in the northernmost colony (Table 1; two-way ANOVA F_3,146_ = 2.39, Tukey post hoc comparisons *p* < 0.05), and the δ^15^N values were significantly lower in chicks than in adults in the two northern colonies (Sites C and D; two-way ANOVA F_3,146_ = 9.85, Tukey post hoc comparisons, both *p* < 0.05).

**Figure 3:**
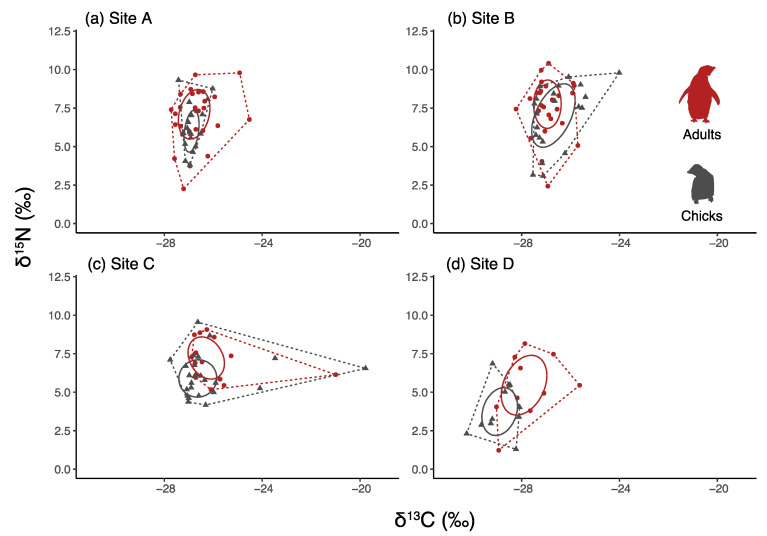
Isotopic niche biplots of adults and chicks in 2017. The polygons represent the TA and the ellipses the SEAc. Adults are represented in red and chicks in black. Letters indicate colonies: (**a**) Site A: Inexpressible Island, (**b**) Site B: Adélie Cove, (**c**) Site C: Edmonson Point, (**d**) Site D: Cape Hallett.

The chicks had narrower isotopic niches than the adults, as indicated by CR, TA and SEAc (Table 1; Figure 3).

**Figure 4:**
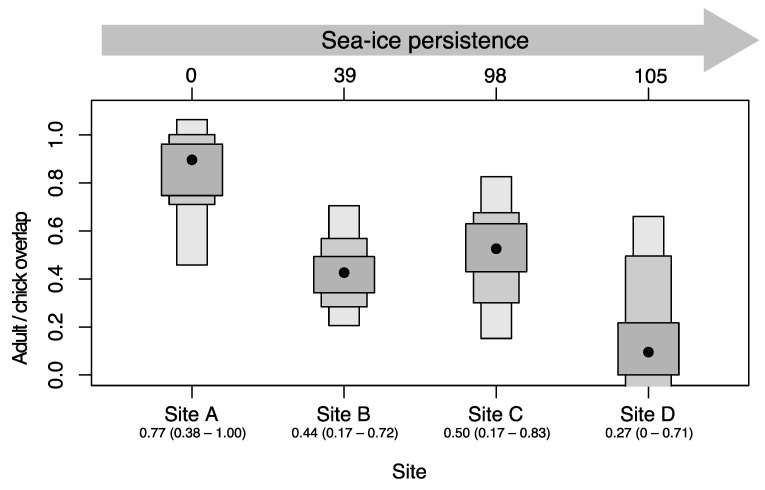
Density plots presenting the Bayesian estimates for the confidence intervals of niche overlap (SEAb overlap) between adults and chicks in each colony in 2016/2017. Solid black circles correspond to the mean Bayesian-estimated overlaps and grey boxes represent the 50%, 75% and 95% confidence intervals. The top bar shows sea-ice persistence (days) in each colony with respect to the polynya (Site A). The longer the sea-ice persistence in the colony, the smaller the niche overlap between adults and chicks. Letters indicate colonies: (A) Inexpressible Island, (B) Adélie Cove, (C) Edmonson Point, (D) Cape Hallett.

The niche overlap (SEAb) was highest near the polynya (Site A) and lowest at the northernmost site (Site D; Figure 4). Notably, in site C, the Carbon Range was wider than in the other colonies in 2017 but decreased significantly in 2018.

### 3.5. The Diet of Adélie Penguins and Its Relationship to Sea-Ice Persistence

Regardless of colony, age or year, penguins did not consume the benthic fish *T. bernacchii* or the benthic-pelagic fish *C. hamatus*, since these fish lay outside the mixing space (Figure S1). Thus, these potential preys were excluded from the mixing models. In addition, due to their similar isotopic values and in accordance with the mixing space model, the pelagic *P. antarctica* and the cryopelagic *P. borchgrevinki* were considered together.

Chicks consumed more krill (Betareg: intercept = 1.18 ± 0.29, z = 4.02, *p* < 0.001, pseudo R^2^ = 0.89) than other resources, including fish (Betareg: coefficient = −2.71 ± 0.44, z = −6.22, *p* < 0.001) and amphipods (Betareg: coefficient = −3.71 ± 0.52, z = −7.18, *p* < 0.001), in all cases, although the exact percentages varied greatly between colonies and between years (Table 4).

**Figure 5:**
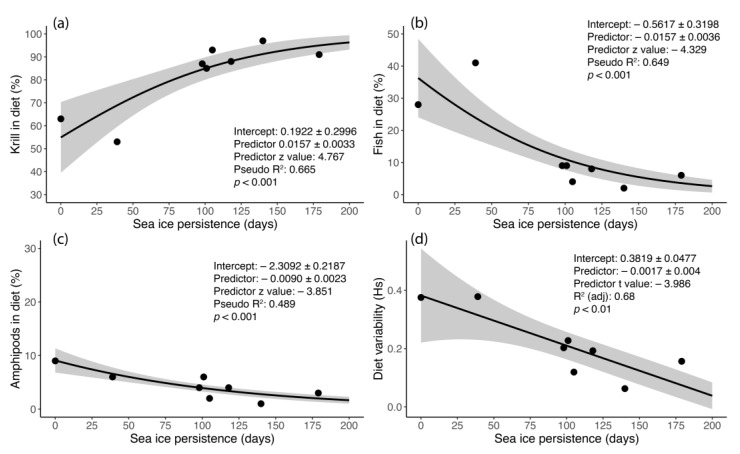
(**a**–**c**) Beta regression models of the effects of sea-ice persistence in 2017 and 2018 on the consumption (percentage of total diet) of (**a**) krill, (**b**) pelagic fish (*Pleuragramma antarctica*) and cryopelagic fish (*Pagothenia borchgrevinki*), (**c**) amphipods. (**d**) Linear regression between sea-ice persistence and the trophic niche width (Hs) of Adélie penguin chicks. Shaded areas show the upper and lower limits of the 95% credibility intervals. The coefficient ± st. error, test statistic, *p* value and adjusted R^2^ are also shown.

The contribution of krill increased markedly from the southern to the northern colonies (Betareg: coefficient = 183.81 ± 91.78, z = 2.00, pseudo R^2^ = 0.95, *p* < 0.05), where the chicks consumed less fish than adults. Krill consumption increased in 2018 (Betareg: coefficient = 1.29 ± 0.16, z = 8.17, pseudo R^2^ = 0.94, *p* < 0.001), above all in the southern colonies (+25%, +32%, +4% and +4% in Sites A, B, C and D, respectively) and in proportion to the delay in sea-ice break-up with respect to 2017 (Table 1). Each day of delay in sea-ice break-up compared to the polynya (site A) determined an increase in krill in the diet, at the expense of fish and amphipods, of about 2% (Figure 5).

Although consumption of amphipods was generally low (mean value estimated by mixing models ≤ 9%) and was negligible for chicks in the northernmost colony (Table 4), some faecal samples had isotopic values very close to those of amphipods (Figure S1), indicating that these individuals had fed almost exclusively on this prey.

## 4. Discussion

In highly dynamic systems such as Antarctica, penguins have developed responses to the space-time heterogeneity of food availability [24,62,89]. For example, Gentoo penguins on the Antarctic Peninsula have been observed to shift their diet towards high-trophic level prey, such as fish, due to the reduction of krill in the area [62,89]. In order to reduce interspecific competition, Adélie and Emperor penguins have developed other food preferences (krill vs. fish) by varying foraging dive distances and depths in response to variation in prey availability [24,62]. Our study highlights sea-ice persistence as a key driver of space-time variability in the diet of Adélie penguin (*Pygoscelis adeliae*) chicks in the Ross Sea.

Due to strong katabatic wind and sea currents, seasonal sea-ice break-up proceeds northwards from the Terra Nova Bay polynya off Inexpressible Island, where sea ice is absent for most of the year, towards Cape Hallett, where sea ice persists longer in summer [29,70,90,91]. This latitudinal sea-ice break-up gradient was measured with reference to satellite images, enabling a landscape-scale assessment of the relationship between sea-ice dynamics and the trophic ecology of Adélie penguin chicks within and between years.

Specifically, the results of this study show that: (1) During summer in the Ross Sea, krill make a larger contribution to the chicks’ diet than fish but in variable proportions depending on the persistence of sea ice. In detail, high consumption of krill was observed when long delays in the sea-ice break-up occurred, almost to the point of saturating the chicks’ diet. (2) Chicks and adults had similar diets, but their isotopic niche diverged as sea-ice persistence increased. The narrower isotopic niche of chicks and the lower δ^15^N of their faeces where the sea ice was more persistent indicate that under those conditions they received less fish than adults.

Regarding the first point, chicks and adults from the northernmost colony, which is not affected by the polynya, had significantly lower δ^13^C and δ^15^N values than chicks and adults from the other colonies. In accordance with other studies’ findings [36,43,50,92], these lower values can be attributed to a higher percentage of krill, since *E. superba* and *E. crystallorophias* have lower δ^13^C and δ^15^N values than the pelagic and cryopelagic fish *P. antarctica* and *P. borchgrevinki*. The increase in krill consumption towards the northern colony, furthest from the polynya, is supported by the results of the Bayesian mixing models. It has also been highlighted in adults by stomach content analysis [23] and more recently by isotopic and DNA-based studies [24,25,27].

While krill made the greatest contribution to the diets of chicks and adults in all colonies, these diets were complemented by pelagic and cryopelagic fish. In contrast, amphipods accounted for only a small proportion, and the contributions of benthic and bentho-pelagic fish were negligible. Although the Adélie penguin is considered a krill-dependent species [93,94], the spatial differences demonstrate that it has great trophic plasticity, since it preferentially feeds on the most readily available prey. The observed dietary differences between colonies were related to the timing of sea-ice break-up, which is known to determine differences in local resource availability. This, in turn, may cause changes in the breeding performance of Adélie penguins, as shown in other studies [95,96]. Indeed, greater abundances of krill have been recorded in years and areas with greater persistence and extent of sea ice [53,97,98,99], while greater availability of *P. antarctica* and *P. borchgrevinki* has been observed in ice-free open waters such as polynyas [20,27,100,101]. The greater consumption of krill than fish where and when ice is more persistent, and particularly in the Edmonson Point and Cape Hallett colonies, is consistent with several studies conducted in the same area. For example, Clarke et al. [102] observed a high consumption of krill at Prydz Bay, East Antarctica. The Prydz Bay colony is often characterised by high persistence of sea ice, which increases the distance to foraging sites and thus prompts penguins to explore a wider resource range (i.e., a more variable diet) and foraging area, as observed in several studies [102,103]. The reduced food availability in this area can make it difficult to obtain sufficient food for chicks, thus reducing the reproductive success of this colony compared to others [102,103].

The enriched δ^13^C and δ^15^N of chicks’ faeces in the southernmost colonies were typical of *E. crystallorophias,* while the more depleted values observed in the northernmost colony were typical of *E. superba* [24]. This is consistent with krill distribution in the Ross Sea, where it has been observed that ice krill (*E. crystallorophias*) is abundant near the southern coast of Terra Nova Bay, in the innermost areas that are more affected by the presence of the polynya, while Antarctic krill (*E. superba*), a pelagic species inhabiting offshore areas, is more common on the northern continental shelf and slope [20,24,27,104,105]. In the case of *E. superba*, a study conducted in East Antarctica by Riaz et al. [106] found in more detail that the diving effort of chick-rearing penguins is concentrated in areas with high swarm numbers rather than high biomass.

Our comparisons between years indicate that sea-ice persistence also determines variability in the trophic ecology of chick penguins over time. More specifically, in the southern colonies (Inexpressible Island and Adélie Cove) the chicks’ faeces had lower isotopic nitrogen values in 2018 than in 2017, which is explained by the lower fish consumption and the increased contribution of krill. This may be associated with the long delay in sea-ice break-up in 2018 compared with 2017. These between-year differences in diet were not observed in the northern colonies (Edmonson Point and Cape Hallett), where sea ice persisted longer than in the other colonies and the diet was based almost exclusively on krill, as also observed by Tabassum et al. [27].

Interestingly, the wide Carbon Range of faeces observed in 2017 in Edmonson Point indicates that chicks relied on two food chains (i.e., pelagic and sympagic), which may have been favoured by the early ice break-up. This is the northernmost colony still under the influence of the polynya and is considered a sink population [46] occupying a transition area between *E. superba* and *E. crystallorophias* [59,107]. By contrast, the much narrower Carbon Range observed in 2018 indicated that at colder temperatures and in more persistent ice conditions, the chicks of this colony had a strictly sympagic-based diet. Further south, in the colonies most affected by the polynya (Inexpressible Island and Adélie Cove), the earlier sea-ice break-up than in the northern colonies increased the probability of encountering cryopelagic fish and krill of the same food chain, as indicated by the narrow Carbon Range and the mixing models.

Regarding the second point, slight differences in δ^15^N values were observed between adults and chicks, indicating the broadly similar composition of their diets. This is consistent with other studies carried out in East Antarctica using both DNA and stable isotope analysis [34,36]. However, differences were more evident where the sea ice was more persistent. Under these conditions, the isotopic niches diverged, and a smaller contribution of fish was observed in chicks than in adults. Fish is considered a more energy-profitable resource than krill [23,34,40], and adults, whenever possible, provide it to their offspring in order to maximise recruitment [31,39]. However, where krill availability is high, chicks are sustained on a krill-based diet [35,108,109]. Low food availability or high population density can prompt parents to take long foraging trips and/or feeding activities at sea when the energy gains exceed the energy costs of obtaining profitable food, as observed by Olmastroni et al. [23,26] when comparing the colonies of Inexpressible Island and Edmonson Point.

The reduced provision of fish to chicks where sea ice is more persistent could be explained by the higher levels of energy spent by adults on longer foraging trips requiring locomotion on the pack ice and swimming in the sea [23,100,110]. In addition, reduced prey availability could result in longer diving activities, increasing both the metabolic rates of adults and the timing of food delivery to chicks [23,46]. In these conditions, adults can optimise energy costs by making separate foraging trips for themselves and their chicks [34] or by assimilating part of the food intended for their offspring to compensate for the energy spent reaching the foraging site [23,26].

The former is supported by the δ^13^C differences between chicks and adults, which can be explained by the specific trophic strategy of adult penguins that prey on resources for themselves and for their offspring in different parts of the sea [36,65]. According to Forero et al. [65], in bird species that feed their chicks, adults carrying food for their offspring in the proventricle tend to partially digest it [36,64,65], and the physical separation of resource collection events is thus the only effective way to separate food for self-maintenance from food for chicks [36,65]. Whether the adults feed their chicks from inshore or offshore waters will depend on the availability of resources [22,34]. While the earlier sea-ice break-up in the two southern sites (Inexpressible Island and Adélie Cove) explains the absence of differences in δ^13^C between adults and chicks, which consume krill and fish from the pelagic chain, in the northernmost colonies, where the sea ice persists longer, the different δ^13^C values suggest consumption of krill from two separate food chains (sympagic vs. pelagic). The results suggest that adults self-feed in coastal waters (inshore) and catch food for their offspring in pelagic waters (offshore). However, the full description of this specific feeding behaviour deserves further investigation by coupling stable isotope analysis of faeces and individual tracking data. Individual tracking will also help clarify the dietary differences observed between individuals. Although outside the scope of this study, differences in the supply of fish with respect to krill may be related to the offspring’s sex, while differences in adult diets can be related to breeders and non-breeders, the latter having no parental costs and no influence on chicks’ diets [34,111].

While our study was limited to the crèche period, sea-ice persistence can have various effects during chick rearing, and the guard period should also be taken into account to understand the effects of sea ice on penguin populations. Indeed, the population dynamics, i.e., growth/survival and chick recruitment, of Adélie penguins are highly complex mechanisms that depend on numerous factors, including rearing, predation and seasonal variation in climatic and sea-ice conditions [26,35]. The direction of changes in sea ice dynamics due to climate change is not uniform across the Antarctic continent and effects are not easy to predict ([46,62] and literature therein). While increasing access to food in the short term, the shortening of the sea ice formation and contraction cycle can result in reduced resource availability for penguins in the long term [112,113]. Reduction or absence of sea ice, linked with the increase in precipitation (snow and rain), could negatively affect this species throughout the year [46]. On the other hand, extensive persistence of sea ice could reduce the survival of penguins by increasing the distances travelled by adults in search of food [102]. Ballerini et al. [46] suggest that an increase in ‘catastrophic’ meteorological conditions could lead to the extinction of the Edmonson Point colony within a few decades and hypothesise that this effect is exacerbated by possible variations in the availability of prey due to sea ice dynamics.

Other parameters such as chick growth and survival can be considered more direct proxies for both assessing the effects of environmental perturbations and understanding whether and how penguin population dynamics are changing [35]. However, quantifying chick diet variation in relation to the timing of sea-ice break-up appears worthy of evaluation, with the purpose of understanding how environmental changes might affect these dynamics in the future by impacting the trophic ecology of the Adélie penguin.

## 5. Conclusions

By quantitatively describing the relationship between krill consumption and sea-ice persistence, this study shows that the space-time variations in the diet and foraging ecology of Adélie penguin chicks in the Ross Sea are determined by variations in sea ice persistence, which is known to be a key driver of resource availability and thus of adult foraging. The contribution of krill to diet decreases when sea-ice break-up occurs earlier. This is important, since krill is a resource not only for penguins but also for other species including pelagic fish, which are alternative prey for the penguins. In contrast, a strong delay in sea-ice break-up could affect the growth and survival of chicks, as the parents may increase their consumption of fish to the detriment of the abundance and quality of food provided to the chicks.

This information adds to our knowledge of the effects of climate on the trophic ecology and reproductive success of the Adélie penguin [44,114]. Climate projections show a strong reduction in sea-ice coverage across Antarctica, with the exception of the Ross Sea, where the trend appears to be the opposite [62]. However, demographic models indicate that in this area too, penguin populations could soon undergo a major decline, becoming functionally extinct within 40 years [46,59], as has already been observed in the Antarctic peninsula [60]. In addition, changes in Adélie penguins’ food preference with climate change over the past 600 years have been shown [62,89].

Stable isotope studies have highlighted an abrupt shift towards lower trophic level prey since the 19th century, which shows that penguins have only recently begun to rely on krill as an important part of their diet [115,116]. The “krill surplus″ hypothesis predicts excessive availability of krill in the Southern Ocean in association with the anthropogenic overexploitation of whales, krill-eating seals and certain fish species. Due to this high availability, krill has become easier to catch than other prey. However, with fish stocks already depleted in the area and penguins having switched to krill in the recent past, declines in the latter prey would leave few foraging opportunities for Adélie penguins in the future.

In the light of these possible variations and due to the potential overlap between this predator’s activity and human fishing, identifying the factors associated with the Adélie penguin’s dietary composition is crucial for the sustainable management of Antarctic marine resources and food webs [3]. The establishment of the “Krill Research Zone” in the Ross Sea, where krill fishing is limited [67,117], will make it possible to preserve krill-consumer populations, including Adélie penguins, and to study the relationships between krill and their predators, revealing the real extent of variations in the Antarctic food web due to climate change and the synergistic effects of human activities.

## Figures and Tables

**Table 1:** Isotopic niche metrics of Adélie penguins (*Pygoscelis adeliae*) and sea-ice persistence. For each of four Adélie penguin colonies in the Ross Sea, the table shows the dates of sea-ice break-up and site-specific sea-ice persistence (days) with respect to the baseline (27 September) in 2016/2017 and 2017/2018. It also shows the delay in sea-ice break-up at each site in the second year of observations with respect to the first year. For Adélie penguin chicks and adults, the table shows the number of samples (n°), δ^13^C and δ^15^N values (Mean ± Standard Error, ‰), carbon range (CR), nitrogen range (NR), total area (TA) and corrected standard ellipse area (SEAc). Letters indicate colonies: (A) Inexpressible Island, (B) Adélie Cove, (C) Edmonson Point, (D) Cape Hallett.

Site	Year	Date of Sea-Ice Break-Up	Sea-Ice Persistence	Sea-Ice Break-Up Delay	Age Class	Sample Size N°	δ^13^C	δ^15^N	CR	NR	TA	SEAc
A	2017	27 September 2016	0		Adults	28	−26.7 ± 0.1	6.9 ± 0.3	5.5	9.7	15	4.1
				118	Chicks	18	−26.8 ± 0.1	6.2 ± 0.3	1.4	5.5	4.3	1.7
	2018	23 January 2018	118		Chicks	18	−27.8 ± 0.2	4.4 ± 0.2	2.7	5.4	9.5	3.7
B	2017	5 November 2016	39		Adults	26	−26.9 ± 0.1	7.5± 0.3	7.5	12	12	3.54
				71	Chicks	21	−26.6 ± 0.2	6.9 ± 0.4	3.5	6.6	12	5.4
	2018	6 January 2018	110		Chicks	20	−26.6 ± 0.2	4.2 ± 0.5	3.5	7.1	14	4.8
C	2017	3 January 2017	98		Adults	15	−25.3 ± 0.4	7.1 ± 0.3	9.1	3.9	20	10.8
				81	Chicks	25	−26.2 ± 0.2	6.0 ± 0.3	7.9	5.3	22	6.9
	2018	25 March 2018	179		Chicks	18	−25.9 ± 0.0	5.4 ± 0.4	1.1	6.4	4.2	1.5
D	2017	10 January 2017	105		Adults	11	−27.8 ± 0.3	5.3 ± 0.4	3.4	6.9	12	6.5
				35	Chicks	10	−28.9 ± 0.2	3.7 ± 0.4	2.1	5.5	7.1	4.1
	2018	14 February 2018	140		Chicks	27	−29.7 ± 0.0	2.9 ± 0.1	1.8	4.5	3.6	1

**Table 2:** Isotopic values of krill (*Euphausia*), amphipods, pelagic fish (*Pleuragramma antarctica*), benthic-pelagic fish (*Chionodraco hamatus*) and benthic fish (*Trematomus bernacchii*) collected during sampling activities. * Isotopic values of cryopelagic fish (*Pagothenia borchgrevinki*) were obtained from the literature (Lauriano et al., 2020). δ^13^C and δ^15^N values are reported as Mean ± Standard deviation (‰). Letters indicate Adélie penguin *(Pygoscelis adeliae)* colonies: (A) Inexpressible Island, (B) Adélie Cove, (C) Edmonson Point, (D) Cape Hallett. TNB indicates Terra Nova Bay. Krill samples at Site D in 2018 were not available (NA).

Prey	Site	Year	δ^13^C (‰)	δ^15^N (‰)	Sample Size
			Mean ± SD	Mean ± SD	N°
Krill	A	2017	−27.99 ± 0.43	5.65 ± 1.25	3
		2018	−28.64 ± 1.17	4.48 ± 0.67	3
	B	2017	−28.03 ± 1.48	6.78 ± 0.49	3
		2018	−27.67 ± 0.50	5.11 ± 0.60	3
	C	2017	−26.31 ± 0.43	7.10 ± 0.88	3
		2018	−25.70 ± 0.50	7.14 ± 0.53	3
	D	2017	−28.08 ± 0.95	5.56 ± 0.32	3
		2018	NA	NA	
Amphipods	TNB	2017−2018	−17.34 ± 0.89	6.13 ± 0.77	14
Pelagic fish (*P. antarctica)*	TNB	2017−2018	−25.54 ± 0.27	10.23 ± 0.29	4
Cryopelagic fish *(P. borchgrevinki)*	TNB		−24.20 ± 0.85	11.50 ± 0.55 *	-
Benthic fish (*T. bernacchii*)	TNB	2017−2018	−22.00 ± 1.08	12.98 ± 0.66	15
Benthic-pelagic fish (*C. hamatus*)	TNB	2017−2018	−24.83 ± 0.98	13.53 ± 1.36	16

**Table 3:** Two-way ANOVA of krill and penguin isotopic values. Effect of colony and year on δ^13^C and δ^15^N values of krill and penguin chicks.

		δ^13^C (‰)		δ^15^N (‰)	
	Factor	F	*p* Value	F	*p* Value
Krill	Site	4.05	0.02	10.47	3.89 × 10^−4^
	Year	0.18	0.68	17.23	6.70 × 10^−4^
	Interaction	0.46	0.72	6.70	3.45 × 10^−3^
Chicks	Site	36.94	7.04 × 10^−18^	11.6	7.13 × 10^−7^
	Year	4.70	0.41	8.80	0.003
	Interaction	1.26	0.29	1.33	0.27

**Table 4:** Resource contributions to the penguin diet. The table shows the percentages (mean, 2.5–97.5% C.I.) of the penguins’ diet accounted for by krill (euphausiids), pelagic/cryopelagic fish (*Pleuragramma antarctica* and *Pagothenia borchgrevinki*) and amphipods in the four colonies: (A) Inexpressible Island, (B) Adélie Cove, (C) Edmonson Point, (D) Cape Hallett.

Prey	Site	Adults 2017	Chicks 2017	Chicks 2018
		Mean% (2.5–97.5% C.I.)	Mean (2.5–97.5% C.I.)	Mean (2.5–97.5% C.I.)
Krill	A	60% (55–74)	63% (54–79)	88% (80–94)
	B	57% (49–65)	53% (41–64)	85% (76–90)
	C	78% (67–93)	87% (81–98)	91% (88–99)
	D	89% (77–97)	93% (82–99)	97% (94–99)
Pelagic/cryopelagic fish	A	31% (26–48)	28% (21–40)	8% (1–16)
	B	40% (30–58)	41% (26–56)	9% (5–17)
	C	14% (2–32)	9% (1–13)	6% (2–18)
	D	7% (1–21)	4% (1–12)	2% (0–5)
Amphipods	A	9% (5–13)	9% (5–12)	4% (2–11)
	B	3% (1–7)	6% (2–13)	6% (1–13)
	C	8% (3–17)	4% (1–10)	3% (0–13)
	D	4% (1–9)	2% (0–8)	1% (0–2)

## Data Availability

Not applicable.

## Supplementary Materials

The following supporting information can be downloaded at https://www.mdpi.com/article/10.3390/biology12050708/s1, Table S1. Post hoc comparisons of the isotopic values of penguin faeces; Figure S1. Simulated mixing regions for Adélie penguin faecal samples (black dots) and potential trophic resources (white dots, mean SD and error bars). Video S1: Sampling Area Sea ice Breakup_nasa-worldview-2016 SEP 27-to-2017 MAR 27; Video S2: Sampling Area Sea ice Breakup_nasa-worldview-2017 SEP 27-to-2018 MAR 27.
