# Supplementary material for: Effects of Sea-Ice Persistence on the Diet of Adélie Penguin (Pygoscelis adeliae) Chicks and the Trophic Differences between Chicks and Adults in the Ross Sea, Antarctica
Source: Biology (Basel). 2023 May 12;12(5):708. doi: 10.3390/biology12050708 (PMC10215695; doi:10.3390/biology12050708)

Article

Effects of Sea-Ice Persistence on the Diet of Adélie Penguin (*Pygoscelis adeliae*) Chicks and the Trophic Differences between Chicks and Adults in the Ross Sea, Antarctica

Deborah Maccapan [1], Giulio Careddu [1,2,*], Edoardo Calizza [1,2], Simona Sporta Caputi [1,2], Loreto Rossi [1,2] and Maria Letizia Costantini [1,2]

1 Department of Environmental Biology, Sapienza University of Rome, Via dei Sardi 70, 00185 Rome, Italy
2 CoNISMa, National Inter-University Consortium for Marine Sciences, Piazzale Flaminio 9, 00196 Rome, Italy
* Corresponding: giulio.careddu@uniroma1.it

Supplementary Material

Table S1. Post-hoc comparisons of the isotopic values of penguin faeces. Post-hoc Tukey multiple comparisons of $\delta^{13}C$ and $\delta^{15}N$ values (95% family-wise confidence level) for penguin chicks in four colonies. P values for comparison are shown in the upper triangular matrix and Tukey's Q is shown in the lower triangular matrix A). Inexpressible Island, B) Adélie Cove, C) Edmonson Point, D) Cape Hallett.

a) $\delta^{13}C$ values of chicks

Colonies	A	B	C	D
A	-	1.00	0.03	0.00
B	0.07	-	0.03	0.00
C	3.74	3.82	-	0.08
D	7.14	7.21	3.39	-

b) $\delta^{15}N$ values of chicks

Colonies	A	B	C	D
A	-	0.51	0.04	0.01
B	2.00	-	0.00	0.00
C	1.90	2.98	-	0.50
D	2.98	4.96	2.00	-

Figure S1. Simulated mixing regions for Adélie penguin faecal samples (black dots) and potential trophic resources (white dots, mean SD and error bars). Colours indicate the probability that the mixing model can explain an individual consumer's isotopic value [72]. Probability contours are at 5% (outermost contour) and every 10% thereafter. Since no consumer point fell near the benthic fish, this resource was excluded from the final mixing.

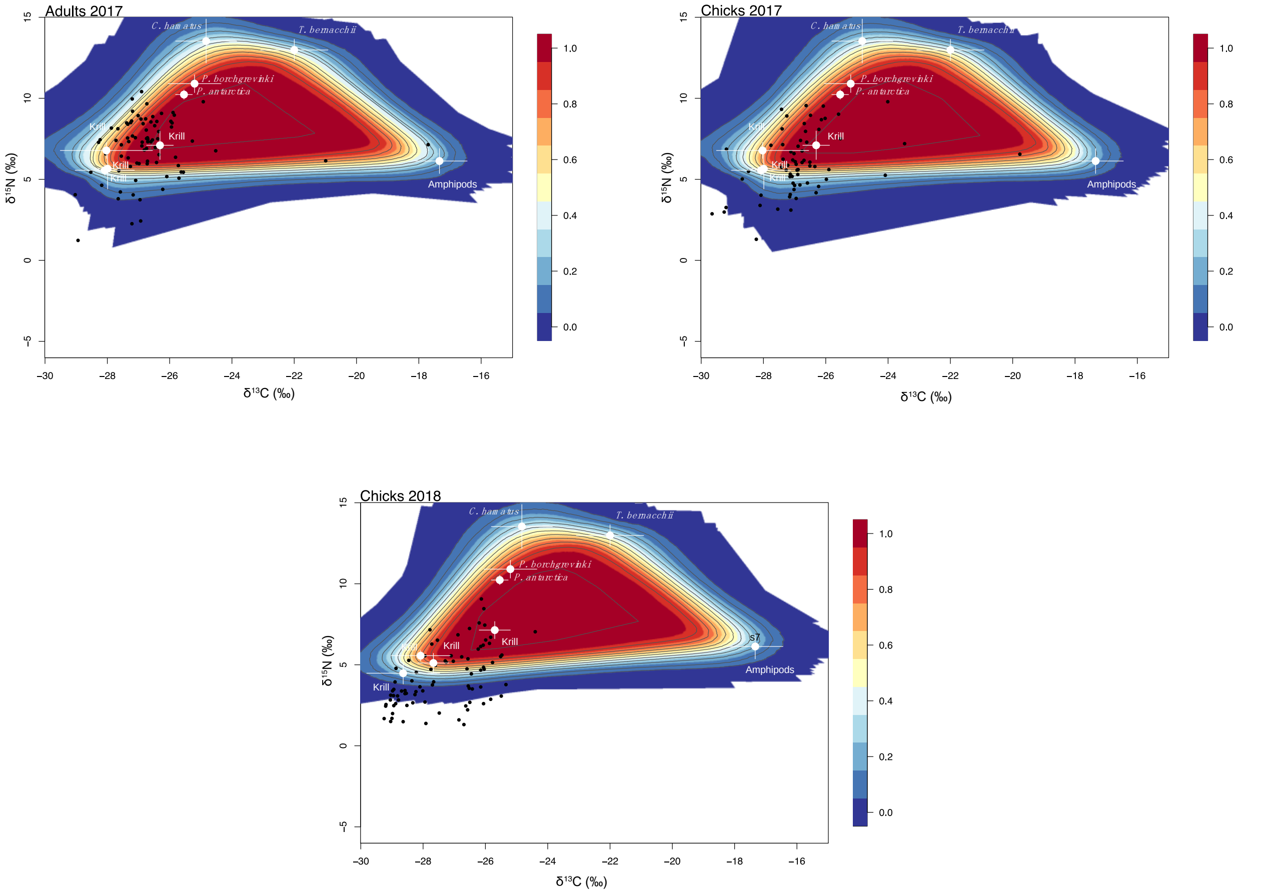

Supplement: Supplementary file 1 [file biology-12-00708-s001.zip › Supplementary Material.pdf]
