# Supplementary figures and images for: Effects of Sea-Ice Persistence on the Diet of Adélie Penguin (Pygoscelis adeliae) Chicks and the Trophic Differences between Chicks and Adults in the Ross Sea, Antarctica
Source: Biology (Basel). 2023 May 12;12(5):708. doi: 10.3390/biology12050708 (PMC10215695; doi:10.3390/biology12050708)

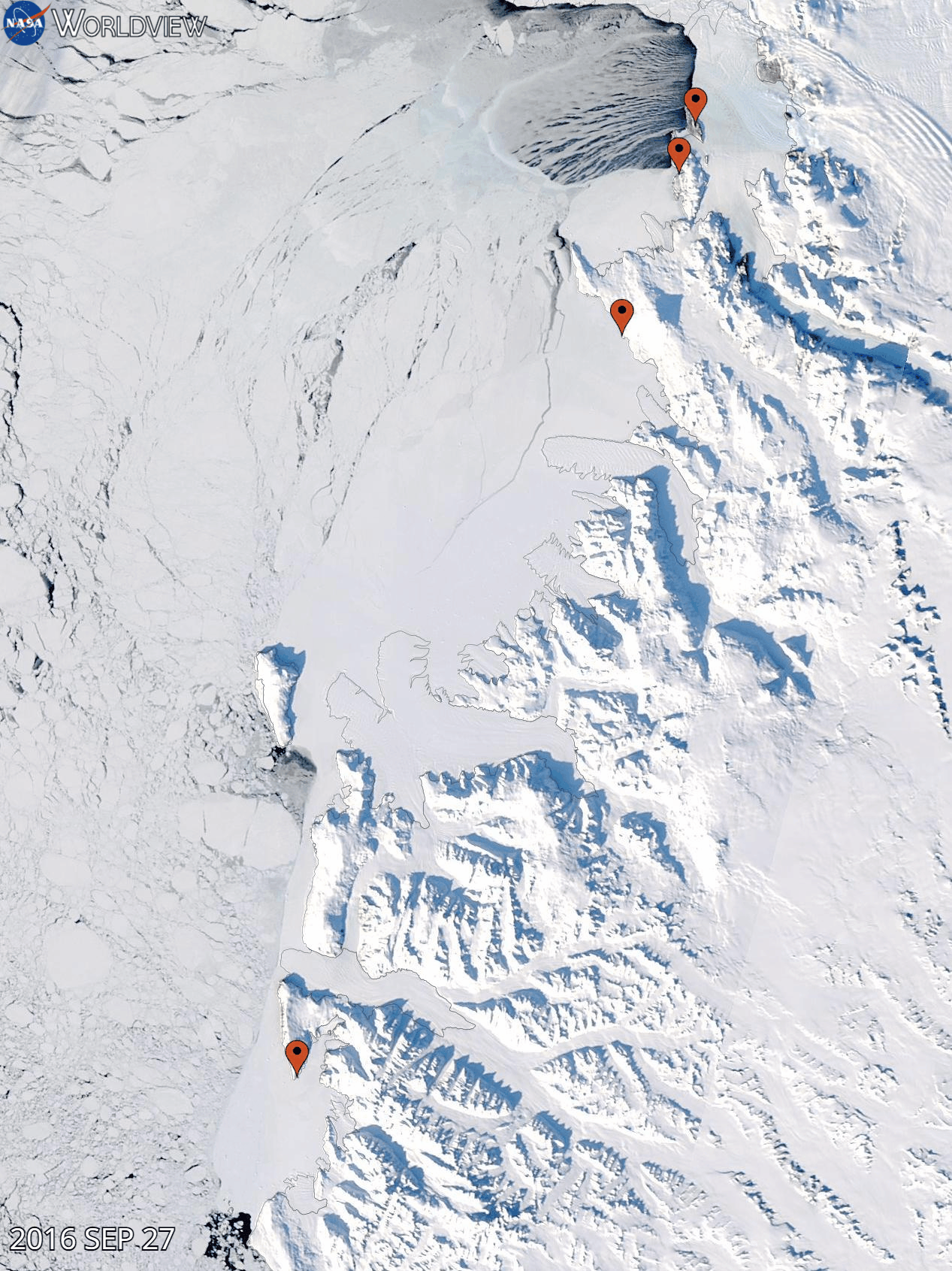

Supplement: Supplementary file 1 [file biology-12-00708-s001.zip › Video S1.gif]
